# Histone acetyltransferase inhibitors block neuroblastoma cell growth *in vivo*

**DOI:** 10.1038/oncsis.2014.51

**Published:** 2015-02-09

**Authors:** J M Gajer, S D Furdas, A Gründer, M Gothwal, U Heinicke, K Keller, F Colland, S Fulda, H L Pahl, I Fichtner, W Sippl, M Jung

**Affiliations:** 1 Institute of Pharmaceutical Sciences, Albert-Ludwigs-University of Freiburg, Freiburg, Germany; 2 Freiburg Institute of Advanced Studies (FRIAS), University of Freiburg, Freiburg, Germany; 3 Section of Molecular Hematology, Department of Hematology/Oncology, University Hospital Freiburg, Freiburg, Germany; 4 Institute for Experimental Cancer Research in Pediatrics, Goethe-University, Frankfurt, Germany; 5 Hybrigenics, 3-5 impasse Reille, Paris, France; 6 German Cancer Consortium (DKTK), Frankfurt, Germany; 7 German Cancer Research Centre (DKFZ), Heidelberg, Germany; 8 Experimental Pharmacology and Oncology Berlin-Buch GmbH, Berlin-Buch, Germany; 9 Department of Pharmaceutical Chemistry, Martin-Luther-University of Halle-Wittenberg, Halle (Saale), Germany; 10 German Cancer Consortium (DKTK), Freiburg, Germany

## Abstract

We have previously described novel histone acetyltransferase (HAT) inhibitors that block neuroblastoma cell growth *in vitro*. Here we show that two selected pyridoisothiazolone HAT inhibitors, PU139 and PU141, induce cellular histone hypoacetylation and inhibit growth of several neoplastic cell lines originating from different tissues. Broader *in vitro* selectivity profiling shows that PU139 blocks the HATs Gcn5, p300/CBP-associated factor (PCAF), CREB (cAMP response element-binding) protein (CBP) and p300, whereas PU141 is selective toward CBP and p300. The pan-inhibitor PU139 triggers caspase-independent cell death in cell culture. Both inhibitors block growth of SK-N-SH neuroblastoma xenografts in mice and the PU139 was shown to synergize with doxorubicin *in vivo*. The latter also reduces histone lysine acetylation *in vivo* at concentrations that block neoplastic xenograft growth. This is one of the very few reports on hypoacetylating agents with *in vivo* anticancer activity.

## Introduction

Reversible protein acetylation on lysine residues is one of the major posttranslational regulatory mechanisms of protein activity. Acetylation sites have been identified in many cellular proteins involved in differentiation and proliferation, signal transduction and metabolism, apoptosis and cytoskeleton dynamics.^1^ Furthermore, histone lysine acetylation is one of the main epigenetic modifications that impact on gene expression and transcriptional activity.^2, 3^ The level of histone lysine acetylation is controlled by the antagonistic catalytic activity of histone acetyltransferases (HATs) and histone deacetylases (HDACs).^4^ In mammalian cells, the HAT family is comprised of three subfamilies: the GNAT^5^ (Gcn5-related *N*-acetyltransferase), the p300/CBP^6^ (CREB (cAMP response element-binding) protein) and the MYST^7^ (Moz, Ybf2, Sas2, Tip60) proteins. All subfamilies include transcription factors as well as steroid receptor co-activators^8^ with catalytic activity.^9, 10, 11^ Because they target both histone and non-histone substrates,^12^ HATs have also been more generally termed lysine acetyltransferases (KATs).^13^

HATs regulate fundamental cell-biological processes and are implicated in the etiology of several diseases. Deregulated HAT activity is particularly linked to cancer formation and progression.^9, 14, 15, 16^ Certain types of leukemia are characterized by the occurrence of fusion proteins with increased HAT activity.^17^ Furthermore, lysine acetylation of the oncogenic fusion protein AML1-ETO by the HAT p300 has been demonstrated in patient blasts using western blotting and is required for leukemic transformation in mouse models as shown by mutation studies. In addition, the p300 inhibitor C646 increased survival in a mouse model of leukemia.^18^ An impaired acetylation equilibrium is also observed in several solid tumors^14, 19^ including prostate,^20, 21^ colon^22^ and breast^23^ cancers with evidences for both HATs and deacetylases as potential drug targets. There is a considerable number of HDAC inhibitors progressing through different stages of preclinical and clinical development and two, namely romidepsin and vorinostat, have already been approved for human use.^24^ In contrast, to date, only a very limited number of HAT inhibitors have been described. Hence, the development and the evaluation of small-molecule HAT inhibitors may provide novel therapeutic approaches.^25, 26^

Several structurally different HAT inhibitors have been reported, including bisubstrate inhibitors,^27, 28, 29, 30^ natural products^31^ and synthetic compounds like isothiazolones containing derivatives^25, 32, 33, 34^ and carboxylic acids.^35, 36^ However, the lack of cell permeability and metabolic stability represent major drawbacks of peptidic inhibitors. Natural products like anacardic acid, curcumin, garcinol and epigallochatechin gallate provide limited mechanistic insight because of a potentially pleiotropic mode of action on a cellular level.^37^ A favorable selectivity profile toward p300/CBP has been reported for the pyrazolone benzoic acid C646^38^ and a very recent report shows that the thiazole CPTH6 induces histone hypoacetylation and apoptosis in leukemic cells.^39^ To date, the investigation of HAT inhibitors has been mostly limited to *in vitro* studies of growth inhibition of cell lines. Only two compounds, the pleiotropic inhibitor sanguinarine that also intercalates DNA^40^ and a water-soluble derivative of curcumin, CTK7A,^41^ have shown histone hypoacetylation in mouse tumor models.

**Figure 1:**
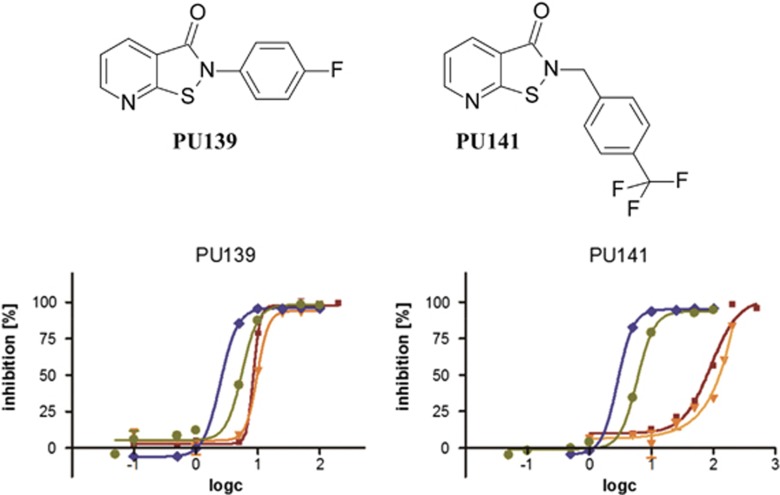
Chemical structures of histone acetyltransferases (HAT) inhibitory *N*-phenyl (PU139) and *N*-benzyl (PU141) pyridoisothiazolones and *in vitro* inhibition of HATs in biochemical assays. Blue lines with diamonds represent inhibition of CBP, olive lines with circles p300, red lines with squares Gcn5 and orange lines with triangles PCAF. The IC_50_ values have been presented in a tabulated version in the literature.^61^

In 2002, the highly reactive isothiazolone core was first reported as a new lead scaffold targeting HAT activities.^34^ Combining *in silico* and *in vitro* screening conducted with the PCAF catalytic domain, we developed pyridoisothiazolones as related HAT inhibitors.^32^ In previous work, we reported *in vitro* HAT inhibitory activity of several compounds with a pyridoisothiazolone scaffold that were identified by virtual screening.^32^ We showed that a series of commercially available pyridoisothiazolones as well as synthesized analogs are potent PCAF inhibitors. They also possess antiproliferative properties, as growth of human neuroblastoma, colon and breast carcinoma cell lines was inhibited. The pyridoisothiazolone class of compounds is known to possess reduced general bioreactivity^42^ as compared with the parent isothiazolones. Nevertheless, the inhibition remains linked to the sulfur–nitrogen bond of the inhibitors that reacts with a cysteine residue within the PCAF-active site. Docking studies suggested favorable positioning of the reactive moiety in close proximity to Cys574.^32^ In accordance with this model, introduction of a methylene linker group led to reduced PCAF inhibition for *N*-benzyl compounds in biochemical assays compared with *N*-phenyl analogs but growth inhibitory properties of the weaker PCAF inhibitors were retained,^32^ implying additional activities, for example, on other HATs. Two pyridoisothiazolones from each compound class, PU139 (*N*-phenyl derivative, strong PCAF inhibition) and PU141 (*N*-benzyl derivative, weak PCAF inhibition; see Figure 1), were selected for further evaluation concerning HAT inhibitory profile and *in vitro* and *in vivo* anticancer properties.

The selectivity profiles were determined for the GNAT and p300/CBP families of HATs. We demonstrate that both inhibitors block tumor growth of SK-N-SH neuroblastoma xenografts. Moreover, the pan-HAT (*N*-phenyl derivative) inhibitor PU139 synergized with doxorubicin *in vivo*. These findings identify pyridoisothiazolones as potent HAT inhibitors with promising anticancer activity *in vivo*.

## Results

### Selectivity profile on HATs

In addition to PCAF, we tested three more recombinant enzymes from the HAT family, namely Gcn5 (KAT2A), CBP (KAT3A) and p300 (KAT3B) for inhibition by PU139 and PU141. Screening was performed using a histone H3 peptide as a substrate- and antibody-based assays with a time-resolved fluorescence readout, commonly known as DELFIA (dissociation-enhanced lanthanide fluorescence immunoassay) technique. Although the *N*-phenyl derivative PU139 was characterized as a potent unselective HAT inhibitor, the *N*-benzyl compound PU141 exhibited CBP/p300 selectivity (Figure 1). The substitution of the isothiazolone nitrogen atom proved to be crucial for the selectivity profile (Figure 1). Thus, despite the covalent and irreversible mode of inhibition, distinct target selectivity can be obtained by modulating the structure of the inhibitor, in this case the relative positioning of the phenyl ring with regard to the pyridoisothiazolone core.

### Off-target selectivity

Although some approved drugs are covalent inhibitors, a general high bioreactivity is undesired because of potential harmful off-target effects. The highly reactive parent isothiazolone core without the annellation had been described to inhibit the cysteine-dependent protease cathepsin B.^43^ To further elaborate on the target selectivity of the inhibitors, we screened other cysteine-containing and -dependent enzymes. In a cysteine protease profiling assay, we included several related enzymes, including caspase 3, a key element of the apoptosis pathway, three ubiquitin-specific processing proteases (UPS 5, 7 and 8) and two ubiquitin carboxy-terminal hydrolases (UCH-L1 and UCH-L3). All enzymes were inhibited by the positive control HBX78273, a potent pan-DUB's (deubiquinating enzymes) inhibitor (Supplementary Figure 1). Our inhibitors PU139 and PU141 were evaluated at eight different concentrations in the range between 200 μM and 91 nM. They did not reveal any significant *in vitro* inhibition on this cysteine protease panel (Supplementary Figure 2) in contrast to the positive control. Hence, despite the cysteine-dependent mode of action, the PU139 and PU141 exert pronounced target selectivity.

### Cytotoxic effects on cancer cells

**Figure 2:**
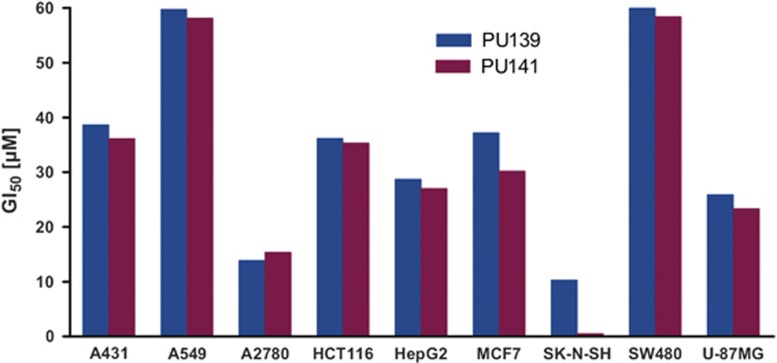
Growth inhibition by PU139 and PU141 on a human cancer cell line panel using a sulforhodamine B (SRB) assay. Cell lines were treated with serial dilutions of the inhibitors (*n*=3). Standard deviation for the shown GI_50_ values did not exceed 10% in each case. A431 (epidemoid carcinoma), A549 (alveolar basal epithelial adenocarcinoma), A2780 (ovarian carcinoma), HCT116 (epithelial colon carcinoma), HepG2 (hepatocellular carcinoma), MCF7 (breast carcinoma), SK-N-SH (neuroblastoma), SW480 (colon adenocarcinoma) and U-87MG (epithelial-like glioblastoma-astrocytoma).

In our previous work, we showed growth inhibitory properties for PU139 and PU141 on SK-N-SH neuroblastoma and MCF7 breast cancer cells.^32^ In order to gain additional knowledge about the cellular activity of pyridoisothiazolones, we investigated the antiproliferative properties on a panel of different human cancer cells. Growth was assessed using a sulforhodamine B cytotoxicity assay for the following solid tumor cell lines: A431 (epidemoid carcinoma), A549 (alveolar basal epithelial adenocarcinoma), A2780 (ovarian carcinoma), HepG2 (hepatocellular carcinoma), SW480 (colon adenocarcinoma), U-87 MG (epithelial-like glioblastoma-astrocytoma), HCT116 (epithelial colon carcinoma) and again SK-N-SH and MCF7 to compare the relative potency in the different assay systems. Both compounds inhibited cell growth at micromolar concentrations in all screened cell lines. The highest cellular antiproliferative activity was detected for the neuroblastoma SK-N-SH cell line (Figure 2). Hence, this cancer type was selected for further inhibitor evaluation *in vitro* and *in vivo* using a xenograft mouse model.

### Induction of caspase-independent cell death

**Figure 3:**
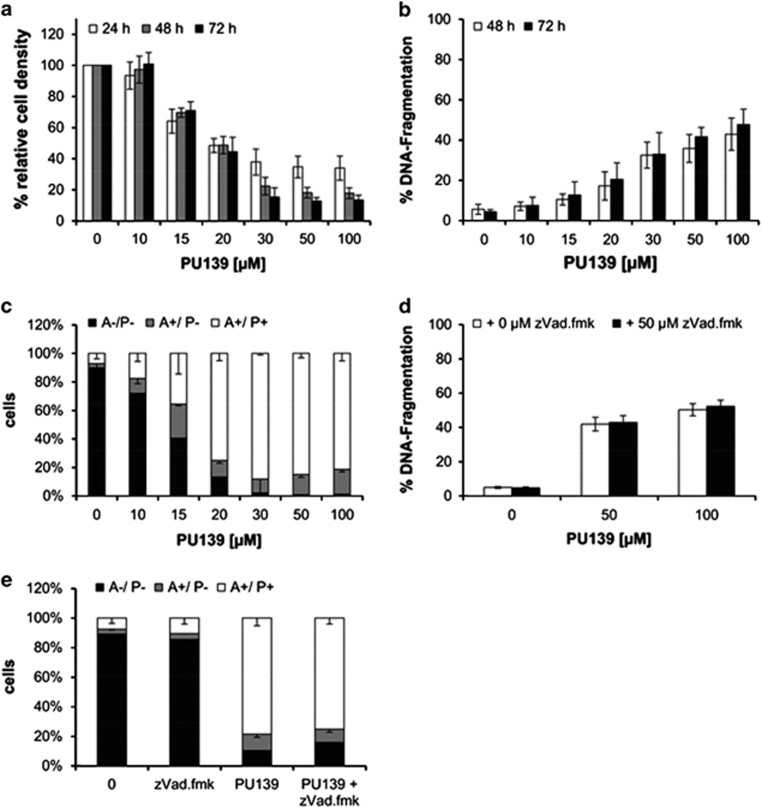
PU139 triggers caspase-independent cell death in the neuroblastoma cell line SK-N-SH. (**a**) SK-N-SH neuroblastoma cells were treated with indicated concentrations of PU139. Relative cell density was measured after 24, 48 or 72 h with crystal violet. (**b**) SK-N-SH cells were treated with the indicated concentrations of PU139. Apoptosis was determined after 48 and 72 h by FACS analysis of DNA fragmentation of propidium iodide (PI)-stained nuclei. (**c**) SK-N-SH cells were treated with the indicated concentrations of PU139. Cell death was determined by Annexin-V/PI staining and FACS analysis. The percentage of Annexin-V-positive/PI double-positive cells is shown. (**d**) SK-N-SH cells were treated with 50 or 100 μM PU139 in the absence and presence of 50 μM zVAD.fmk. Apoptosis was determined after 72 h by FACS analysis of DNA fragmentation of PI-stained nuclei. (**e**) SK-N-SH cells were treated with 20 μM PU139 in the absence and presence of 50 μM zVAD.fmk. Cell death was determined after 72 h by Annexin-V/PI staining and FACS analysis. The percentage of Annexin-V-positive/PI double-positive cells is shown. All experiments were shown as mean and s.d. of three experiment performed in triplicates.

To determine both the cytostatic and cytotoxic activities of PU139, we performed a crystal violet assay, as SK-N-SH cells detach from the surface when they undergo cell death. Treatment with PU139 substantially reduced cell density in a dose-dependent manner (Figure 3a). To investigate whether this reduction of cell density involves the induction of apoptosis, we analyzed both DNA fragmentation and phosphatidylserine exposure on the cell surface by Annexin-V staining as two characteristic parameters of apoptotic cell death. Treatment with PU139 resulted in a moderate increase in DNA fragmentation at concentrations that profoundly reduced cell density (Figure 3b). Similarly, concomitant analysis of Annexin-V/propodium iodide staining revealed that PU139 caused only a slight increase in Annexin-V single-positive cells, whereas it strongly enhanced the amount of Annexin-V/propidium iodide (PI) double-positive cells (Figure 3c).

As these data suggest that cell death following PU139 treatment is not primarily mediated by the induction of apoptosis, we tested whether caspases are involved in PU139-induced cell death by using the broad-range caspase inhibitor zVAD.fmk. The addition of zVAD.fmk failed to protect against PU139-induced DNA fragmentation (Figure 3d). In addition, zVAD.fmk did not substantially alter the percentage of Annexin-V single-positive or Annexin-V/PI double-positive cells after treatment with PU139. In contrast, zVAD.fmk significantly reduced the percentage of Annexin-V single-positive cells upon treatment with the HDAC inhibitor JNJ-26481585 that was used as a reference control (Supplementary Figure 3) Together, this set of experiments indicates that PU139 triggers caspase-independent cell death.

### Histone hypoacetylation

**Figure 4:**
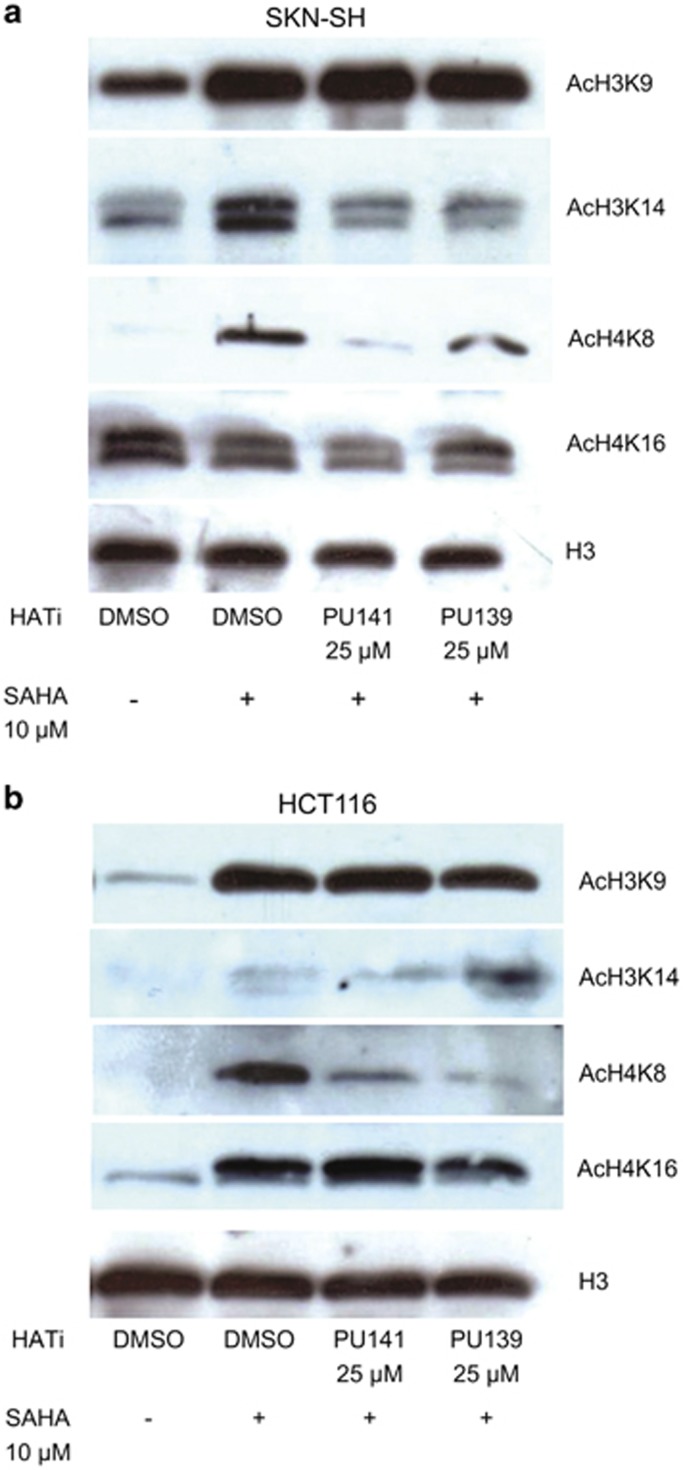
Histone acetylation levels of PU139- and PU141-treated SK-N-SH neuroblastoma and HCT116 colon carcinoma cells. Cells were treated with 25 μM of the HAT inhibitors and 10 μM SAHA. Cells treated with 10 μM SAHA alone and dimethyl sulfoxide (DMSO) as a vehicle were used as controls. After an incubation time of 3 h, cells were harvested and histones were acid-extracted overnight. Acetylation levels were detected using antibodies against acetylated Histone H3K9, K3K14, H4K8 and H4K16, respectively. An antibody directed against the C-terminus of the unmodified Histone H3 (Active Motif, Belgium) served as a loading control.

Histone acetylation levels were studied in PU139- and PU141-treated SK-N-SH and HCT116 cells. In general, basal histone acetylation is low for many lysine residues, which impairs detection of hypoacetylation as a consequence of HAT inhibition. Therefore, cells were co-treated with the HDAC inhibitor SAHA (suberoylanilide hydroxamic acid, vorinostat) in order to increase basal acetylation and to generate a larger window for the detection of hypoacetylation. Antibodies specifically directed against acetylated lysine residues on H3 and H4 were utilized for detection in western blotting experiments. SAHA did lead to hyperacetylation as compared with solvent control. Both compounds led to a decrease in SAHA-induced H3K14 and H4K8 hyperacetylation, whereas H3K9 and H4K16 acetylation levels remained stable after co-treatment of HDAC and HAT inhibitor (Figure 4). The impact on histone acetylation was similar in both SK-N-SH and HCT116 cells. In addition, using a pan-acetyl histone H3 antibody, we observed histone hypoacetylation in HL60 leukemia cells (see Supplementary Figure 4). Thus, we demonstrate that our HAT inhibitors cause both histone hypoacetylation and growth inhibition (Figure 2) *in vitro*.

### Synergy with doxorubicin

In clinical trials, epigenetic inhibitors have often shown weak efficacy when used as single agents.^44^ Hence, combination therapy might prove to be the key to successful implementation of epigenetic inhibitors in cancer treatment. The anthracycline doxorubicin is a well-characterized DNA-intercalating drug that is used in the therapy of neuroblastoma, combined with cisplatin, etoposide and cyclophosphamide (CEDC regime).^45^ Modulation of histone acetylation levels by HDAC inhibitors led to increased cytotoxicity of doxorubicin *in vitro*.^46, 47, 48, 49^ We therefore hypothesized that doxorubicin may also synergize with HAT inhibitors and tested this on SK-N-SH cells *in vitro.*^50^ Indeed, combining doxorubicin with the pan-inhibitor PU139 led to slightly synergistic effects on the suppression of viability (Supplementary Figure 5).

### Neuroblastoma xenograft model

The neuroblastoma cell line SK-N-SH that responded best to HAT inhibitor treatment in cell culture was then used for *in vivo* evaluation of the anticancer activity of PU139 and PU141. A xenograft model was established in male NMRI:nu/nu mice. The compounds were administered once intraperitoneally (i.p.) as a detergent containing saline microemulsion. Initial studies were conducted to determine a tolerable concentration range using concentrations from 25 to 100 mg/kg body weight per injection and identified 25 mg/kg as maximum tolerated dose.

**Figure 5:**
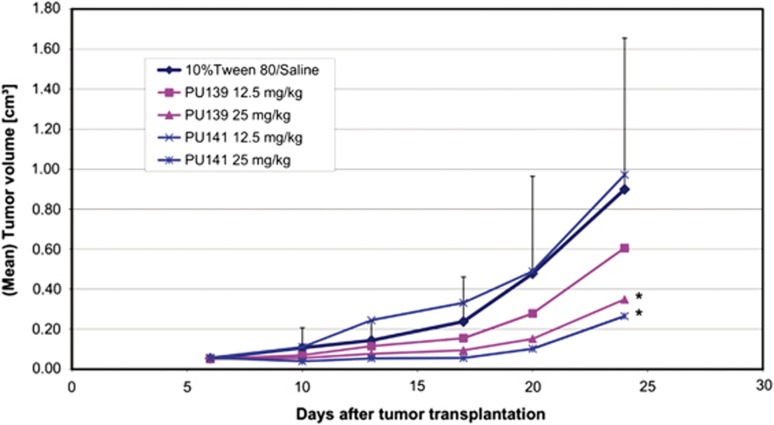
Comparison of the anticancer activity of PU139 and PU141 in a neuroblastoma xenograft NMRI mouse model. Each mouse received at day zero one fragment of the neuroblastoma SK-N-SH (taken from an *in vivo* passage) subcutaneously into the left flank. When tumors were growing mice were randomized to the treatment groups (eight mice per arm) and treatment was initiated. Both test compounds were solubilized in 10% Tween-80 in saline and administered once per week i.p. Tumor size was measured twice per week and individual tumor volumes (TV) were calculated according to length × width^2^/2. Mean values of each treatment group were related to the control group and *T*/*C* values in % were determined. At the end of each experiment, mice were killed and tumors were taken for further analyses. For statistical comparisons, the *U*-test of Mann and Whitney was used with a significance level of *P*<0.05 (Statistica 5.0). *Signifcant to control, *P*<0.05.

To compare the antitumoral efficacy of PU139 and PU141 among each other, eight male NMRI:nu/nu mice received one neuroblastoma fragment each from *in vivo* passage subcutaneously and were monitored for 24 days in comparison to the saline control group. Compound administration was undertaken on days 6 and 13. Blood parameters were checked on day 10, while tumor volume and body weight were evaluated twice a week. Both pyridoisothiazolones led to significant tumor volume reduction (33% and 19% for PU139 and PU141, respectively) at 25 mg/kg (Figure 5). Minimal loss in weight (1%) was detected for both inhibitors (Supplementary Figure 6). Blood parameters were not altered for all cohorts (data not shown). These findings provided evidence for the *in vivo* activity of pyridoisothiazolones as anticancer agents.

### Doxorubicin synergy

In a subsequent cohort of mice, we compared the effect of PU139 at 25 mg/kg i.p. with the antitumoral activity of doxorubicin at 8 mg/kg i.v. Drugs were administered on days 14 and 21 as a single dose treatment of each compound or, for combination therapy; both drugs were administered successively within 1 h. Mice in a cohort were killed when tumors reached a mean size of 1.2 cm^3^, according to the animal welfare plan.

**Figure 6:**
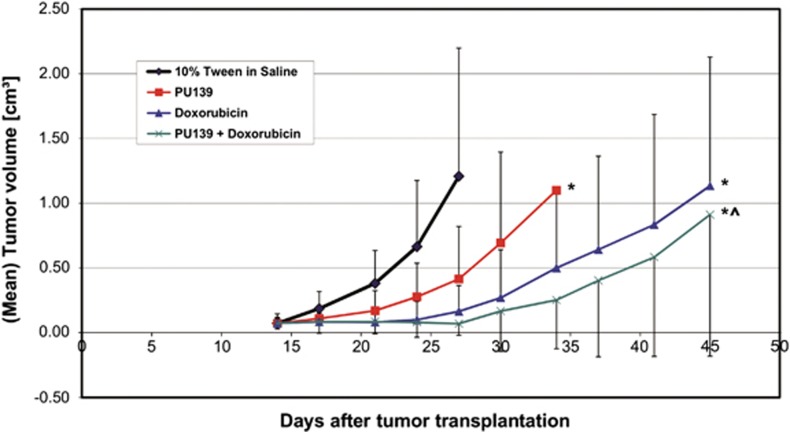
Comparison of the anticancer activity of doxorubicin and PU139 administration as a single and co-treatment in a neuroblastoma xenograft model. Experiments were performed as described in Materials and methods and legend to Figure 5. Doxorubicin (Pfizer, Karlsruhe, Germany) was dissolved as prescribed. The control group received 10% Tween-80 in saline (* signifcant to control, *P*<0.05; ^ significant to single treatment, *P*<0.05).

During the whole experimental period, the observed body weight changes were tolerable, comprising 11% loss of body weight for Doxorubicin alone and 16% for the combination. Doxorubicin alone led to a strong and significant tumor volume reduction (13% *T*/*C*). Optimum growth inhibition following a single PU139 therapy was moderate (34% *T*/*C*), but significant as compared with the untreated group and confirmed the previous findings. More important, treatment with both doxorubicin and HAT inhibitor induced enhanced antitumoral effects (6% *T*/*C*) that were significantly stronger than those seen in the administration of either agent alone (Figure 6).

### *In vivo* acetylation in healthy mice

**Figure 7:**
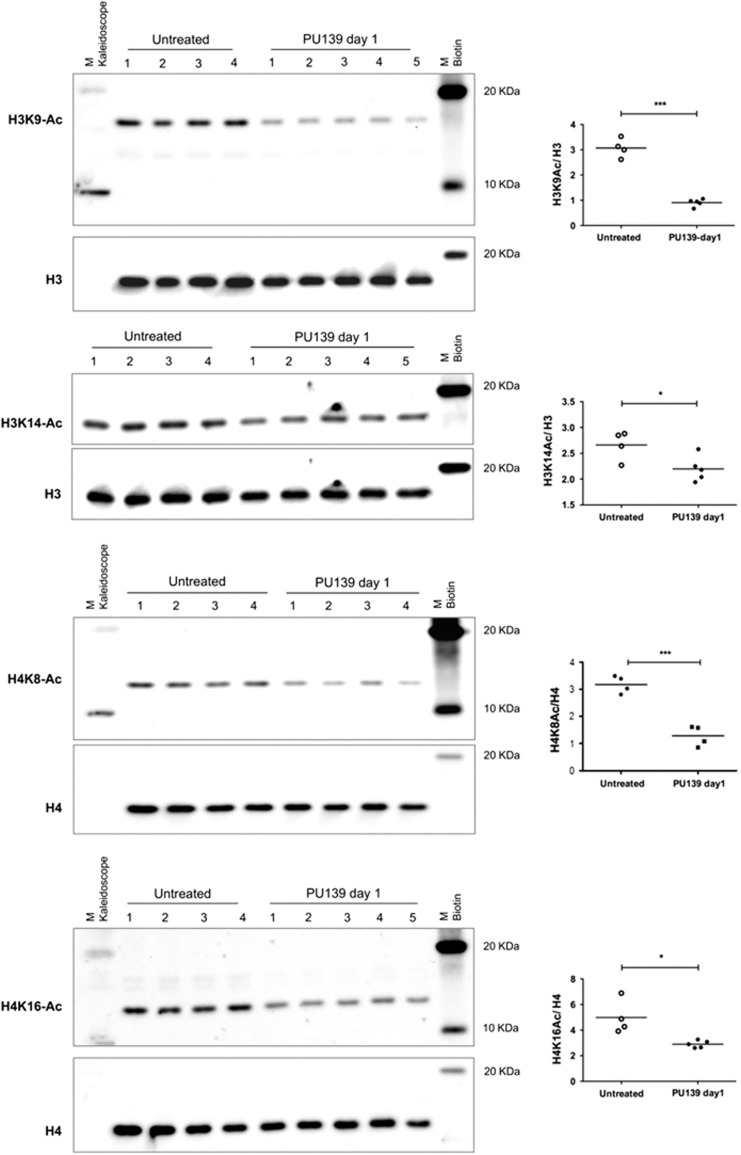
Histone acetylation levels of PU139-treated healthy NMRI:nu/nu mice. Mice were injected intraperitoneally with 100 μl PU139 (10 mg/ml, 25 μg/g body weight). Bone marrow cells were flushed in Iscove's Modified Dulbecco's Medium (IMDM) medium using a 26-G syringe on day 1 post PU139 injections. Red blood cell lysis was carried out with erythrocyte lysis buffer (Sigma) followed by centrifugation and resuspension of the pellet in IMDM. Cell counting was done using a Neubauer counting chamber. For western blot analysis, cells were resuspended in IMDM containing SDS loading dye and 10 mM sodium butyrate. Typically, 10^5^ cells were lysed in 100 μl IMDM. To check acetylation levels, lysates were probed with antibodies against H3K14-Ac, H4K16-Ac, H3K9-Ac and H4K8-Ac (* signifcant to control, *P*<0.05; *** significant to control, *P*<0.001).

To link target engagement to the growth inhibitory activity, we subsequently analyzed the histone acetylation levels in xenografts at the end of the observation period. No changes were detected. We hypothesized that this is due to transient effects caused by the HAT inhibitors and the long time elapsed after the last administration of drugs. We therefore investigated the effect of PU139 in short-time exposure experiments. Healthy NMRI:nu/nu mice, the same strain used for xenografting, were injected with 25 mg/kg PU139 i.p., the effective dose both as a single agent and in combination with doxorubicin, and analyzed 24 h later. Significant hypoacetylation was detected on histone H3K9, H3K14, H4K8 and H4K16. There is a difference to the cultured tumor cells where mostly H3K14 and H4K8 were affected (Figure 7). By contrast, methylation levels on H3K9me3 and H3K27me3 were unaltered by PU139 treatment, demonstrating specificity of the inhibition (Supplementary Figure 7).

## Discussion

Even though histone acetylation is one of the major mechanisms of epigenetic regulation, relatively little is known about the therapeutic potential of HAT inhibitors. This is in stark contrast to the well-studied effects of HDAC inhibitors, although both HATs and HDACs are involved in the control of the acetylation status of histones. Although several small molecules targeting HDACs are currently subject to clinical evaluation and two have gained Food and Drug Administration approval,^16^ only a few compounds with HAT inhibitory properties have been characterized *in vivo* so far.^26^ Several natural products have been investigated mostly in combination with established drugs.^51^ However, owing to the pleiotropic mode of action of natural compounds, it is difficult to link their phenotypic responses specifically to their HAT inhibitory activity. This highlights the demand for new and more specific HAT inhibitors.

In response to this need, we set out to develop and characterize novel HAT inhibitors. The isothiazolone core is known to target several cellular proteins among them farnesyltransferases,^52^ telomerase^53^ and p65^lck^ tyrosine kinase.^54^ This is due to the high general bioreactivity of the chemical scaffold, which is caused by the presence of the cysteine-reactive sulfur–nitrogen bond.^34^ The annellation of the isothiazolone core to a pyridine moiety resulting in the compounds used in this study led to inhibitors with retained target spectrum toward HATs and reduced general bioreactivity and increased target selectivity. This was confirmed by a lack of inhibition of five different DUBs as well as caspase 3, all cysteine-depending enzymes.

Within the HAT family, the *N*-phenyl inhibitor PU139 is a potent pan-HAT inhibitor, targeting Gcn5, PCAF, p300 as well as CBP. On the other hand, PU141 from the *N*-benzyl class emerged as a CBP/p300-selective inhibitor. In GNAT enzymes, the presence of a methylene linker in the *N-*benzyl compound PU141 probably impedes optimal positioning due to an extended conformation within the active site of GNAT enzymes. Unlike GNAT, the catalytic pocket of p300/CBP is characterized by a wider binding cavity.^6^ Consequently, a large number of substrates have been reported to bind p300/CBP transiently and undergo acetylation.^38^ These structural features of the KAT3 enzymes explain the CBP/p300 selectivity of the *N*-benzyl derivative PU141.

Hypoacetylation has been investigated on lysines that are known to be modified preferably by Gcn5 and PCAF (H3K9 and H3K14)^27^ and CBP (H3K14, H4K8 and H4K16)^55^ *in vitro*. Antiproliferative properties for PU139 and PU141 on a panel of human cancer cell lines and reduced histone acetylation levels (H3K14 and H4K8) show target engagement in the same dose range in which the phenotypic response was observed. This demonstrates a clear correlation between the HAT inhibition *in vitro* and the antiproliferative activity of these compounds in cellular assays. Importantly, both the pan-HAT inhibitors PU139 and the KAT3-selective inhibitor PU141 exhibited a significant antitumor effects against neuroblastoma xenografts *in vivo.* In addition, PU139 was shown to synergize with doxorubicin used as a prototypic chemotherapeutic drug in growth inhibition *in vivo*. This points out to a potential use of HAT inhibitors in combination chemotherapy. For HDAC inhibitors, many such combinations are currently studied in clinical trials^44^ and a systematic analysis of synergies of HAT inhibitors with other epigenetic agents but also other classic anticancer drugs is warranted. Furthermore, PU139-treated healthy mice showed significant histone hypoacetylation in bone marrow on all four investigated lysine residues in a short-time exposure experiments confirming target engagnement *in vivo*. The observed difference to the cultured tumor cells where mostly H3K14 and H4K8 were affected could be tissue specific. No impact on histone methylation (H3K9 and K27me3) has been found *in vivo* implicating specific effects on histone acetylation. Altoghether, these findings reveal a novel therapeutical approach based on a small-molecule epigenetic modifier targeting HATs. Further optimization of subtype selectivity, potency and pharmacokinetic properties, for example, solubility in water, should provide new potential anticancer drugs.

## Materials and methods

### Inhibitors

Inhibitors (PU139, PU141, SF7, SF18 and SF19) were synthesized using already published procedures.^32^ Besides full spectroscopic characterization of the compounds, purity was monitored by high-performance liquid chromatography and determined to be over 98%. If not differently mentioned, assays were performed using dimethyl sulfoxide solutions of the inhibitors stored at –20 °C that were prepared newly at regular intervals.

### HAT assays

Heterogeneous assays based on the DELFIA technology were performed for *in vitro* screening of HAT inhibitory activities as reported previously for PCAF (KAT2B; *n*=3).^32^ The PCAF recombinant catalytic domain used for testing was prepared according to the published protocol for bacterial overexpression of the His-tagged fusion protein.^36^ Gcn5 (KAT2A; *n*=2) was purchased from BPS Bioscience (catalog#50070, San Diego, CA, USA), CBP (KAT3A; *n*=2) from Biomol (catalog # SE-452, Hamburg, Germany), p300 (KAT3B; *n*=2) from BPS Bioscience (catalog # 50071).^32^ Dimethyl sulfoxide was used as solvent control.

### Cysteine proteases profiling assay

USP7, 5, 8 and UCH-L1, 3 were purified as previously described^56^ and diluted in deubiquitinating enzyme buffer (50 mM Tris-HCl, 0.5 mM EDTA, 5 mM DTT, 0.01% Triton X-100, bovine serum albumin 0.05 mg/ml^−1^; pH 7.6 for all enzymes with the exception of USP8: pH 8.8). Caspase 3 was diluted in caspase 3 buffer (100 mM HEPES, 10% sucrose, 0.1% 3-[(3-cholamidopropyl)dimethylammonio]-1- propanesulfonate (CHAPS); pH 7.5). Compound stocks (10 mM in dimethyl sulfoxide) were stored at –20 °C. Compounds were tested at eight different concentrations (from 200 to 91 nM). Reactions were performed as duplicates in Black 384-well plates (Greiner Bio-one, Courtaboeuf Cedex, France) with 10 μl final reaction volumes.

The substrate concentration for all deubiquitinating enzymes was 300 nM Ub-AMC^57^ (Boston Biochem, Cambridge, MA, USA). The substrate concentration for caspase 3 specificity assay was 250 nM Ac-DEVD-AMC (Promega, Mannheim, Germany). The following enzyme concentrations were used in specificity assays: USP7 (100 pM), USP5 (300 pM), USP8 (1.36 μM), UCH-L1 (2.5 μM), UCH-L3 (12.8 pM) and caspase 3 (1.6 μM). The concentrations were initially determined for specificity assays under initial velocities at a fixed substrate concentration. Compounds were pre-incubated with enzymes for 30 min at 25 °C. Reactions were initiated by addition of substrate to the plates containing the enzymes (±compounds) diluted in assay buffer. Reactions were incubated for 60 min at 37 °C and stopped with acetic acid (100 mM final concentration). Readings were performed on a PHERAstar Fluorescent Reader (BMG Labtech, Ortenberg, Germany). *λ*_EM_: 380 nm; *λ*_EX_: 460 nm. Data (mean values±standard deviation) were analyzed as a percentage of control (no compound) and plotted versus the Log of the compound concentration using GraphPad (Prism). Data were fitted to a sigmoidal model (variable slope).

### Cell viability and cell death assays

Data from cytotoxicity screening with SK-N-SH, HCT116, MCF7, A431, A549, A2780, HCT116, HepG2, SW480 and U87-MG cells were obtained with sulforhodamine B assay as established in the literature^58, 59^ by Biosolutions (Halle, Germany). After initial determination of the compound-specific concentration range for each cell line and pyridoisothiazolone, serial dilutions were prepared for PU139 and PU141. GI_50_ values were calculated applying the SigmaPlot software (*n*=3). Standard deviation for the shown GI_50_ values did not exceed 10% in each case.

Cell death was assessed by Annexin-V/PI staining (Roche, Grenzach, Germany) and flow cytometry according to the manufacturer's instructions. Apoptosis was determined by analysis of DNA fragmentation of PI-stained nuclei using flow cytometry. Cytotoxicity was determined by crystal violet assay using crystal violet solution (0.75% crystal violet, 50% ethanol, 0.25% NaCl, 1.57% formaldehyde). The HDAC inhibitor JNJ-26481585 was used as a positive control. The broad-spectrum caspase inhibitor *N*-benzyloxycarbonyl-Val-Ala-Asp-fluoromethylketone (zVAD.fmk; Bachem, Heidelberg, Germany) was used to test the involvement of caspase activity.

### Analysis of synergism with doxorubicin *in vitro*

For the combination studies of the *N*-phenyl-derivative with the cytostatic agent doxorubicin, SK-N-SH neuroblastoma cells were treated with three different concentrations (25, 50 and 75 nM) of doxorubicin and varying concentrations of the HAT inhibitor for 72 h. GI_50_-values were determined using the CellTiter 96 Aqueous Non-Radioactive Cell Proliferation Assay as described previously.

The Chou–Talalay method^60^ was used for the evaluation. It uses the following equation: CI=(*D*)1/(*Dx*)1+(*D*)2/(*Dx*)2, for which (*D*)1 and (*D*)2 are respective doses of drugs 1 and 2 that have the effect *x* used in combination, and (*Dx*)1 and (*Dx*)2 are the doses of drugs 1 and 2 that have the same effect when used alone. Synergy is present when cooperativity index (CI)<1.0. The combination has additive effects when CI=1.0, and is antagonistic when CI>1.0.

### Histone acetylation levels

SK-N-SH neuroblastoma and HCT116 colon carcinoma cells were treated with 25 μM of the HAT inhibitors and 10 μM SAHA. Cells treated with 10 μM SAHA alone and dimethyl sulfoxide as a vehicle were used as controls. After an incubation time of 3 h, cells were harvested and histones were acid-extracted over night. The proteins were separated using a 15% SDS–polyacrylamide gel electrophoresis and transferred to a Polyvinylidene fluoride (PVDF)-membrane (Roti-PVDF, Carl Roth, Karlsruhe, Germany). Acetylation levels were detected using antibodies against acetylated Histone H3K9, K3K14, H4K8 and H4K16, respectively (all Active Motif, La Hulpe, Belgium). An antibody directed against the C-terminus of the unmodified Histone H3 (Active Motif) as a loading control.

### Neuroblastoma xenografts

For the *in vivo* experiments, male NMRI:nu/nu mice purchased from Charles River (Sulzfeld, Germany) were used. Each mouse received at day zero one fragment of the neuroblastoma SK-N-SH (taken from an *in vivo* passage) subcutaneously into the left flank. When tumors were growing (50–80 mm^3^) mice were randomized to the treatment groups (eight mice per arm) and treatment was initiated. Both test compounds were solubilized in 10% Tween-80 in saline and administered once per week i.p. Doxorubicin (Pfizer, Karlsruhe, Germany) was dissolved as prescribed. The control group received 10% Tween-80 in saline. Tumor size was measured twice per week and individual tumor volumes (TV) were calculated according to length × width^2^/2. Mean values of each treatment group were related to the control group and *T*/*C* values in % were determined. Body weight was determined twice per week, mean values per group were calculated and related to the first treatment day (body weight change, BWC in %). Hematological parameters were determined 3–4 days after initiation of treatment. At the end of each experiment, mice were killed and tumors were taken for further analyses. For statistical comparisons, the *U*-test of Mann and Whitney was used with a significance level of *P*<0.05 (Statistica 5.0). All animal experiments were approved by the local responsible authorities (LaGeSo Berlin, A0452/08).

### *In vivo* acetylation levels in healthy mice

PU139 was dissolved in a solution of Tween-80/0.9% NaCl (1:10). Mice were injected i.p. with 100 μl PU139 (10 mg/ml, 25 μg/g body weight). Bone marrow cells were flushed in IMDM medium using a 26-G syringe on day 1 post PU139 injections. Red blood cell lysis was carried out with erythrocyte lysis buffer (Sigma) followed by centrifugation and resuspension of the pellet in IMDM. Cell counting was done using a Neubauer counting chamber. For western analysis, cells were resuspended in IMDM containing SDS loading dye and 10 mM sodium butyrate. Typically, 10^5^ cells were lysed in 100 μl IMDM. Cells were sheared by passing the cells five or six times through a 26-G 1-ml syringe, immediately followed by boiling at 95 ^o^C for 5 min. Lysates were stored at −20 ^o^C until use. To check acetylation levels, lysates were probed with antibodies against H3K14-Ac (Active Motif), H4K16-Ac (Active Motif), H3K9-Ac (Active Motif) and H4K8-Ac (Cell Signaling, Danvers, MA, USA). In addition, western blot analysis was done for H3K27me3 (Millipore, Billerica, MA, USA) and H3K9me3 (Millipore). Blots were stripped and reprobed with antibodies against total H3 (Abcam, Cambridge, UK) or total H4 (Abcam) to ensure equal loading.
