# Supplementary material for: Histone acetyltransferase inhibitors block neuroblastoma cell growth in vivo
Source: Oncogenesis. 2015 Feb 9;4(2):e137–. doi: 10.1038/oncsis.2014.51 (PMC4338425; doi:10.1038/oncsis.2014.51)
Supplement: Supplementary Information [file oncsis201451x1.pdf]

Histone acetyltransferase inhibitors block neuroblastoma cell growth *in vivo*

[1]JM Gajer[#], [1,2]SD Furdas[#], [3]A Gründer, [3]M Gothwal, [4]U Heinicke, [1]K. Keller, [5,10]F Colland, [4]S Fulda, [3]HL Pahl, [6]I Fichtner, [2,7]W Sippl and [1,8,9]M Jung*

[#]These authors contributed equally to the manuscript.

[1]Institute of Pharmaceutical Sciences, University of Freiburg, Freiburg Germany

[2]Freiburg Institute of Advanced Studies (FRIAS), University of Freiburg, Freiburg, Germany

[3]Section of Molecular Hematology, Department of Hematology/Oncology, University Hospital Freiburg, Freiburg, Germany

[4]Institute for Experimental Cancer Research in Pediatrics, Goethe-University, Frankfurt, Germany

[5]Hybrigenics, 3-5 impasse Reille, 75014 Paris, France

[6]Experimental Pharmacology & Oncology Berlin-Buch GmbH, Berlin-Buch, Germany

[7]Department of Pharmaceutical Chemistry, Martin-Luther-University of Halle-Wittenberg, Halle (Saale), Germany

[8]German Cancer Consortium (DKTK), Freiburg, Germany

[9]German Cancer Research Centre (DKFZ), Heidelberg, Germany

[10]Current address: Oncology Research & Development Unit, Institut de Recherches Servier, 125 Chemin de ronde, 78290 Croissy sur Seine, France

Correspondence: Prof. Dr. Manfred Jung, Institute of Pharmaceutical Sciences
Albert-Ludwigs-Unversity of Freiburg, Albertstr. 25, 79104 Freiburg, Germany, Tel: +49-761-203-4896, Fax: +49-761-203-6321
e-mail: manfred.jung@pharmazie.uni-freiburg.de

Supplementary Figures

Supplementary Figure S1

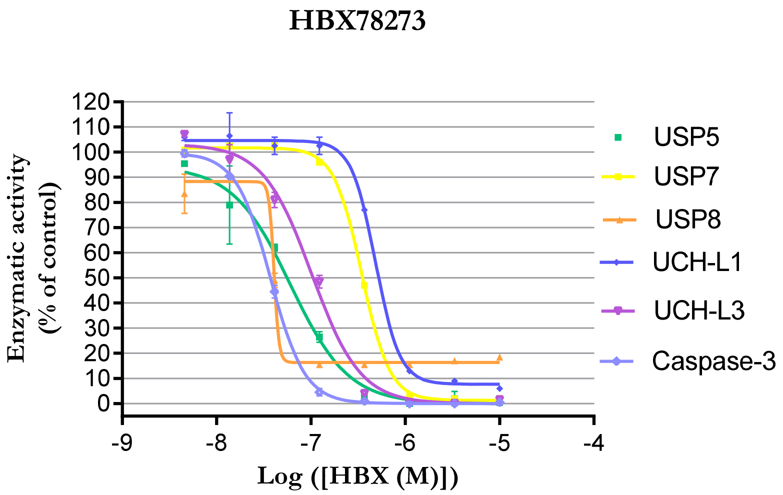

	USP5	USP7	USP8	UCH-L1	UCH-L3	Caspase-3
EC50	6.087e-008	3.465e-007	~ 4.068e-008	4.852e-007	1.020e-007	3.732e-008

Supplementary Figure S1. *In vitro* inhibition of the reference inhibitor HBX78273 on a cysteine protease panel. The tested enzymes are ubiquitin-specific processing proteases (UPS5=Hgx932, USP7=Hgx682, and USP8=Hgx777), ubiquitin carboxy-terminal hydrolases (UCH-L1 and UCH-L3), and caspase 3. The assay was started after pre-incubation of HBX78273 with the enzymes for 30 minutes at 25 °C by addition of substrate. Inhibition was determined after incubation for 60 minutes at 37 °C. All enzymes were inhibited by HBX78273 (EC50, M)

Supplementary Figure S2

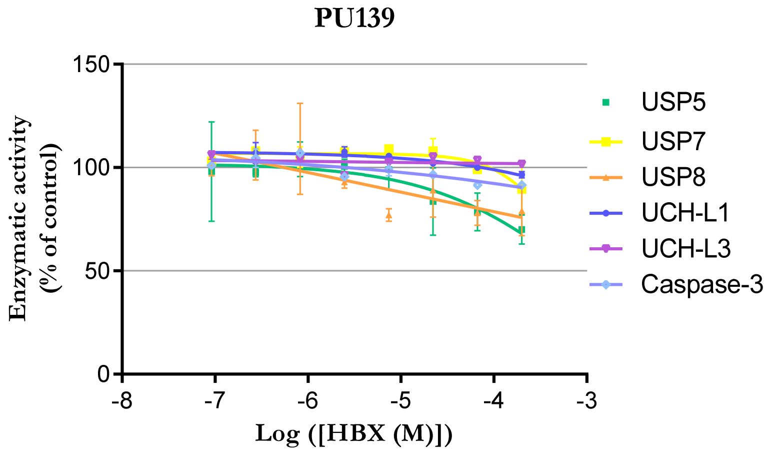

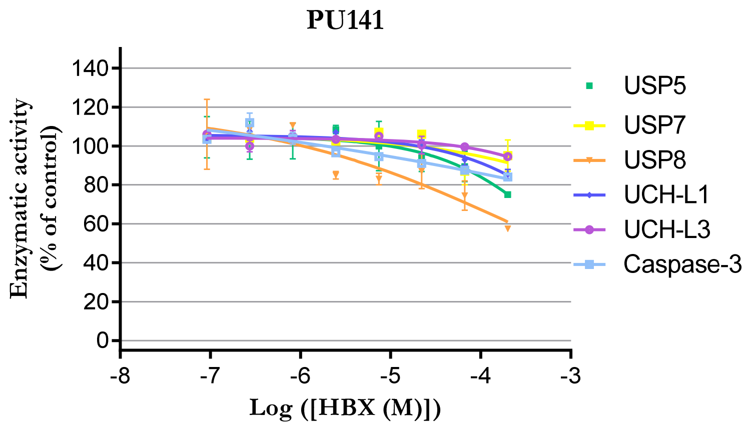

Supplementary Figure S2. *In vitro* inhibition of PU139 and PU141 on a cysteine protease panel. The tested enzymes are ubiquitin-specific processing proteases (UPS5=Hgx932, USP7=Hgx682, and USP8=Hgx777), ubiquitin carboxy-terminal hydrolases (UCH-L1 and UCH-L3), and caspase 3. The enzymes were pre-incubated with eight different concentrations of PU139 and PU141. Inhibition was determined after incubation with substrate for 60 minutes at 37 °C. Even above 100 µM inhibition was less than 30 % in most cases and not more than 40% in any case.

Supplementary Figure S3

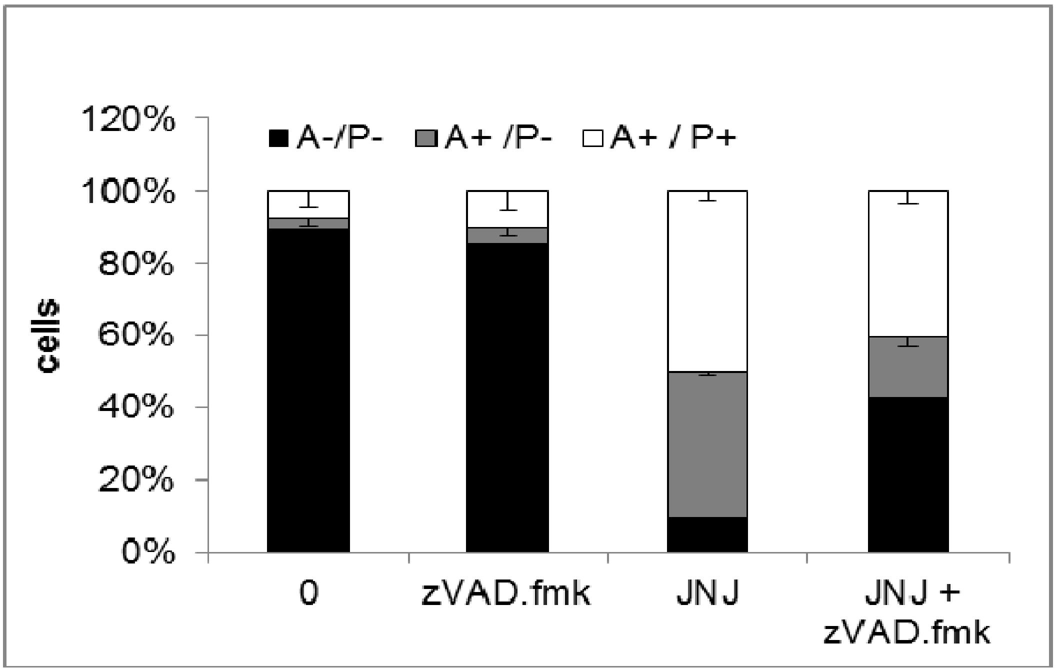

Supplementary Figure S3. The HDACi JNJ-26481585 triggers caspase-dependent cell death in the neuroblastoma cell line SK-N-SH. SK-N-SH cells were treated with 50 nM JNJ-26481585 in the absence and presence of 50 µM zVAD.fmk. Cell death was determined after 48 hours by Annexin-V/PI staining and FACS analysis. The percentage of Annexin-V positive/PI double positive cells is shown. zVAD.fmk reduced the percentage of Annexin-V single positive cells when treated with JNJ-26481585. All experiments are shown as mean plus/minus SD of three experiments performed in duplicates.

Supplementary Figure S4

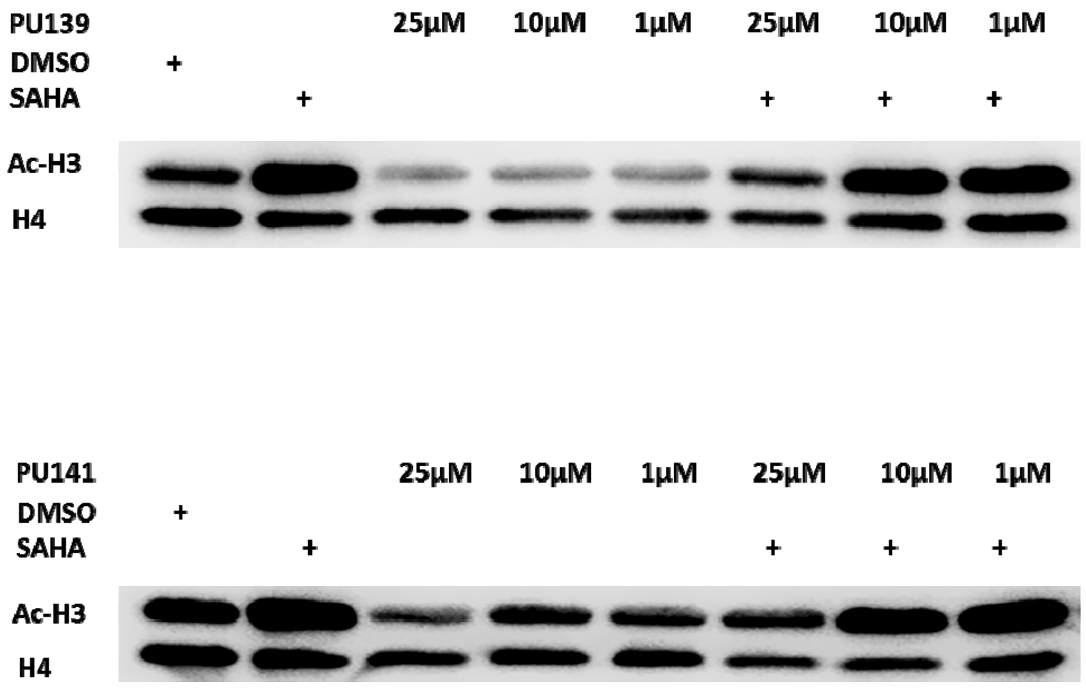

Supplementary Figure S4. Histone hypoacetylation in HL60 leukemia cells after treatment with PU139 and PU141. DMSO: solvent control. SAHA: addition of the HDAC inhibitor SAHA (10 µM in all cases) which increases acetylation to produce a larger window of effect. Cells were incubated for 3 h with 10 µM SAHA and different concentrations of PU139 and PU141. Cells were harvested and histones were acid-extracted overnight. Histone hypoacetylation in HL60 cells could be detected for both inhibitors with regard to DMSO control as well as compared to SAHA hyperacetylated controls.

Supplementary Figure S5

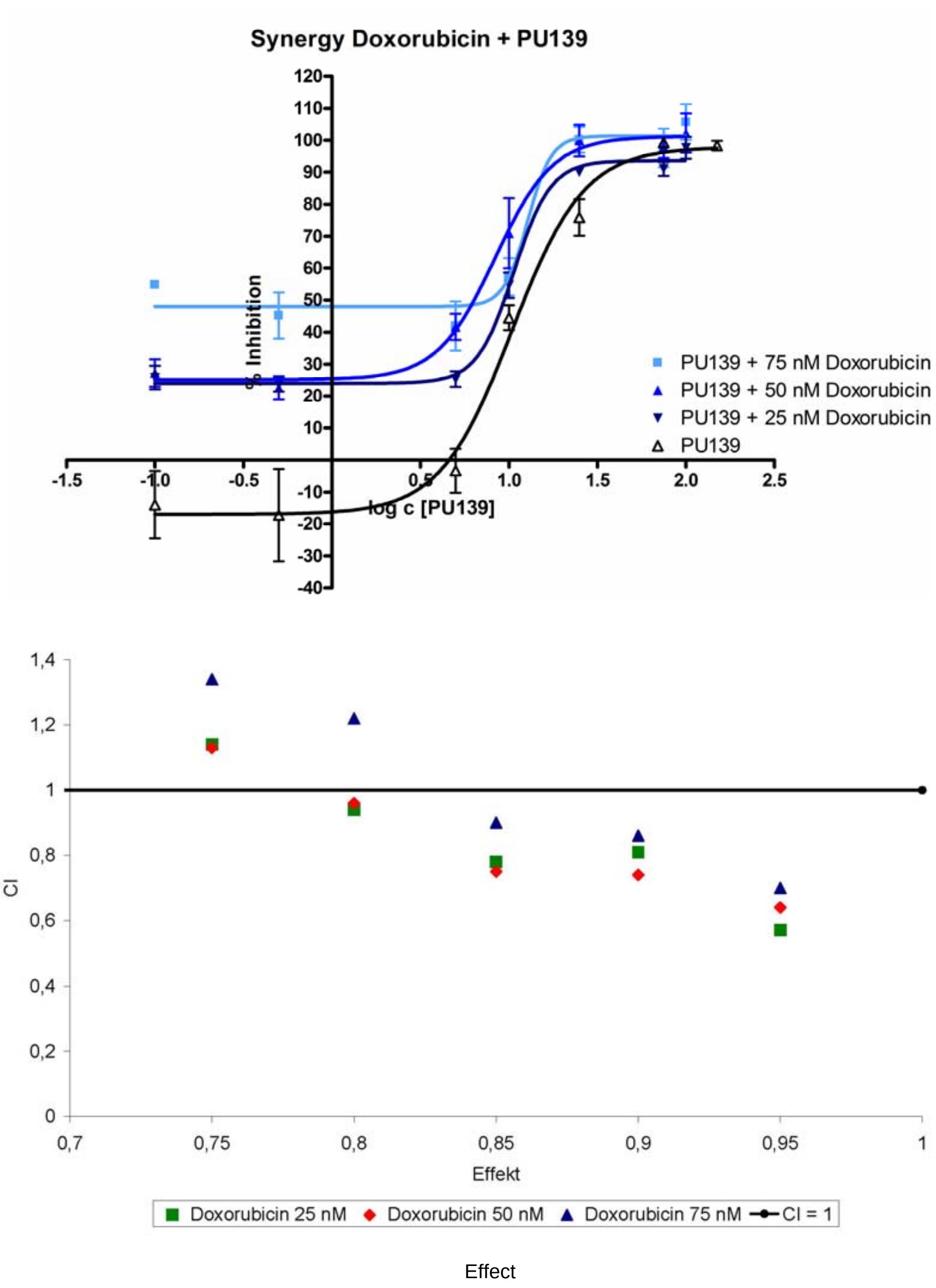

Supplementary Figure S5. Synergy of doxorubicin and PU139 on cellular viability. Cells were incubated for 72 h with three different concentrations of Doxorubicin and varying concentrations of PU139. Dose response curves (upper panel) and calculated CI value (lower panel) are shown. The combination has synergistic effects when CI<1.0, additive effects when CI=1.0 and antagonistic effect when CI>1.0.

Supplementary Figure S6

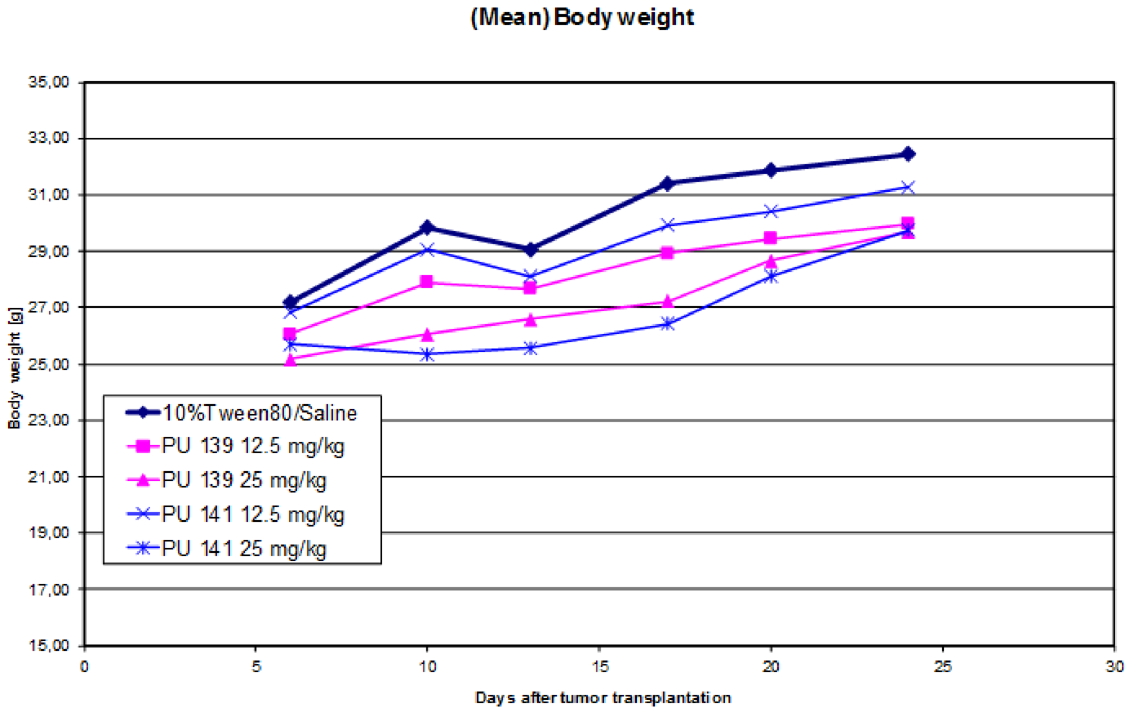

Supplementary Figure S6. Body weight curves during treatment with PU139 and PU141. Body weight was determined twice per week and mean values per group were calculated. Minimal loss of weight could be observed for both PU139 and PU141.

Supplementary Figure S7

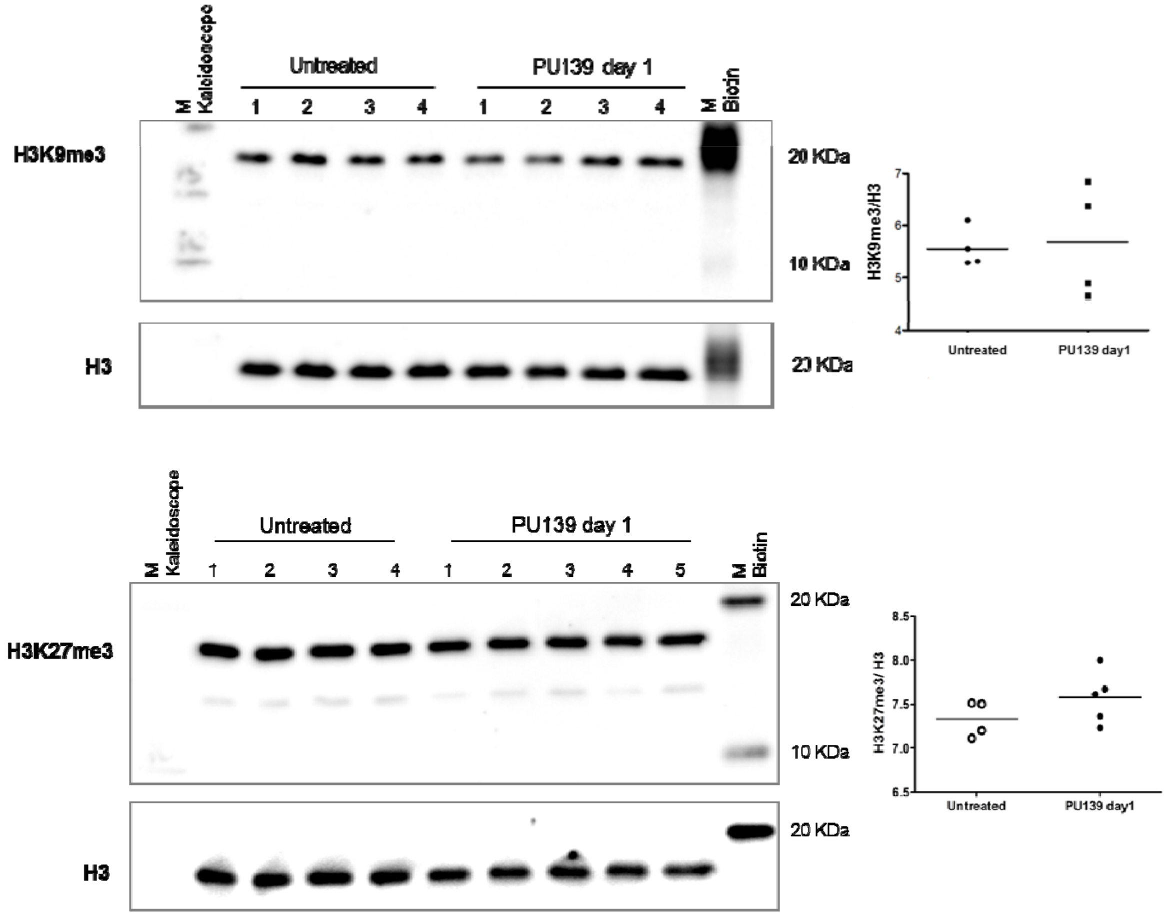

Supplementary Figure S7. Histone methylation levels of PU139 treated healthy NMRI mice. Bone marrow cells were flushed on day one post PU139 injection. Red blood cells were destroyed by erythrocyte lysis buffer. 10^5 cells per 100 µl were lysed and methylation levels were analysed. PU139 shows no effect on H3K9me3 (upper panel) and H3K27me3 (lower panel).
